# Meta-analysis of colorectal cancer follow-up after potentially curative resection

**DOI:** 10.1002/bjs.10233

**Published:** 2016-08-04

**Authors:** S Mokhles, F Macbeth, V Farewell, F Fiorentino, N R Williams, R N Younes, J J M Takkenberg, T Treasure

**Affiliations:** Department of Cardio-Thoracic Surgery, Erasmus University Medical Centre, Rotterdam, The Netherlands; Wales Cancer Trials Unit, Cardiff University, Cardiff, UK; Medical Research Council Biostatistics Unit, Institute of Public Health, University of Cambridge, Cambridge, UK; Division of Surgery and Cancer, and Imperial College Trials Unit, Imperial College London, London, UK; Surgical and Interventional Trials Unit, Division of Surgery and Interventional Science, Faculty of Medical Sciences, University College London, London, UK; Oncology Centre, Hospital Alemão Oswaldo Cruz, Sao Paulo, Brazil; Clinical Operational Research Unit, University College London, London, UK

## Abstract

**Background:**

After potentially curative resection of primary colorectal cancer, patients may be monitored by measurement of carcinoembryonic antigen and/or CT to detect asymptomatic metastatic disease earlier.

**Methods:**

A systematic review and meta-analysis was conducted to find evidence for the clinical effectiveness of monitoring in advancing the diagnosis of recurrence and its effect on survival. MEDLINE (Ovid), Embase, the Cochrane Library, Web of Science and other databases were searched for randomized comparisons of increased intensity monitoring compared with a contemporary standard policy after resection of primary colorectal cancer.

**Results:**

There were 16 randomized comparisons, 11 with published survival data. More intensive monitoring advanced the diagnosis of recurrence by a median of 10 (i.q.r. 5–24) months. In ten of 11 studies the authors reported no demonstrable difference in overall survival. Seven RCTs, published from 1995 to 2016, randomly assigned 3325 patients to a monitoring protocol made more intensive by introducing new methods or increasing the frequency of existing follow-up protocols *versus* less invasive monitoring. No detectable difference in overall survival was associated with more intensive monitoring protocols (hazard ratio 0·98, 95 per cent c.i. 0·87 to 1·11).

**Conclusion:**

Based on pooled data from randomized trials published from 1995 to 2016, the anticipated survival benefit from surgical treatment resulting from earlier detection of metastases has not been achieved.

## Teaser

Does it really help?

## Introduction

A variety of monitoring strategies have been used in patients who have had potentially curative surgery for primary colorectal cancer. Their aim has been to detect active disease before it is symptomatic or clinically evident so that further treatment can be instigated. Five randomized trials published from 1995 to 1998 were the subject of a systematic review and meta-analysis published in 2002^1^. Intensive follow-up was associated with significantly earlier detection by a mean of 8·5 months. The combined risk ratio was 0·81 (95 per cent c.i. 0·70 to 0·94) in favour of intensive follow-up. However, the authors found that methods were poorly reported and concluded that ‘large trials are required to identify which components of intensive follow up are most beneficial’. Since then, three large trials^2–4^ of intensified monitoring have reported. An updated search, systematic review and meta-analysis have been undertaken to examine the effect of these programmes on overall survival including all randomized studies identified.

## Methods

A systematic review of literature on follow-up strategies for patients with colorectal cancer was conducted according to the PRISMA guidelines^5^ and is registered in PROSPERO (CRD42015026835). This study was based on predefined eligibility criteria and conducted according to a predefined methodological approach.

### Search strategy

An extensive search for published articles was conducted in collaboration with a medical librarian. The electronic databases of MEDLINE (Ovid), Embase, the Cochrane Library and Web of Science, Scopus, CINAHL (EBSCO), PubMed publisher, Google Scholar, LILACS, SciELO and ProQuest were searched. The searches identified four index terms: large intestinal cancer, surgery, periodical surveillance and mortality or survival. Appropriate thesaurus terms (for MEDLINE, Embase and CINAHL) and keywords in the title and/or abstract were combined by Boolean logical operators, and adapted to the appropriate syntax of each database. The reference lists of reviews and included studies were cross-checked.

### Selection of studies

Papers were screened by two independent investigators, arbitrated by a third reviewer. Data were extracted from studies reporting randomly assigned groups of patients in surveillance protocols of differing intensity. Only studies conducted in humans and written in English were included. Studies with inadequate data on survival for meta-analysis were retained for textual summaries of the design, findings and conclusions.

### Outcome measures

The primary outcome was the overall survival difference between the existing monitoring strategy compared with a more intensive monitoring strategy.

### Quality control

Studies were checked independently for quality using the Cochrane risk of bias tool^6^. The authors of all studies were approached for further information^7^.

### Data extraction

Data were extracted by one researcher and checked independently by a second reviewer. A third investigator resolved any discrepancies. Patient numbers, baseline characteristics, all-cause mortality, cancer-specific mortality and recurrence rates were retrieved for each study.

Overall survival data were extracted as event rates reported for more *versus* less intensive monitoring arms of all randomized comparisons. Odds ratios (ORs) and their variances were calculated. Hazard ratios (HRs) were derived from Kaplan–Meier curves. The method described by Williamson and colleagues^8^ was used to estimate a logarithmic HR with corresponding variance when the number of patients at risk was given at each time point. If these data were not provided, the method of Parmar *et al*.^9^ was used. The overall HR with 95 per cent c.i. was estimated using an inverse variance-weighted average^10^.

### Statistical analysis

Review Manager (RevMan) for Windows® version 5.3 (Nordic Cochrane Centre, Cochrane Collaboration, Copenhagen, Denmark) was used for meta-analysis. Funnel plots were used to investigate publication bias. Heterogeneity among the included studies was analysed by means of the *I*^2^ measure^11^. A random-effects meta-analysis was performed after exclusion of trials with a high risk of bias.

ORs were also used to summarize observed effects, and a random-effects logistic regression model was used to provide an overall estimate of an effect for subsets of studies defined by the chosen method of enhanced detection. Subgroup analyses of outcome were performed to account for different diagnostic tests used during follow-up in different randomized trials. Studies were grouped as follows: any site of recurrence; endoscopically detected recurrence; or the clinical setting of follow-up. Sensitivity analyses were performed to identify studies that were estimated to have a high risk of bias.

Meta-analysis was undertaken and forest plots were constructed for all trials that reached the criteria for inclusion. Since the previous meta-analysis calling for large trials^1^, there have been three large multicentre trials, published in 2006, 2014 and 2016^2–4^ relating to policies of earlier detection of patients suitable for the growing practice of metastasectomy^12–14^. The analysis was repeated in this subset of trials.

## Results

**Fig. 1:**
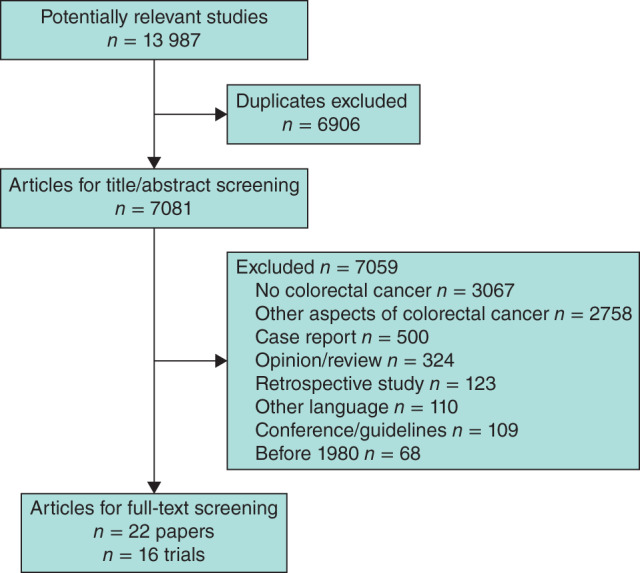
Flow chart showing selection of trials for review

**Fig. 2:**
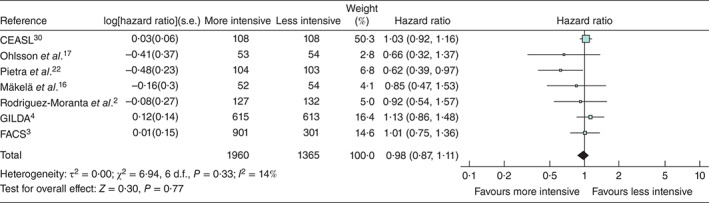
Forest plot showing hazard ratios for death in seven randomized comparisons of more and less intensive follow-up from which hazard ratios could be derived. An inverse-variance random-effects model was used to produce an overall estimated hazards ratio. Hazard ratios are shown with 95 per cent confidence intervals. The studies are ordered according to the year of the start of the inclusion. CEASL is dominant because the weight of the study is dependent on the follow-up time, number of events and number of patients in each treatment arm. The Kaplan–Meier curve in CEASL is plotted up to 25 years. The point estimate in favour of more intensive monitoring in studies by Rodriguez-Moranta *et al.*^2^ and Pietra and colleagues^22^ was attributed by the authors to detection by endoscopy and successful treatment of recurrent rectal carcinoma

**Fig. 3:**
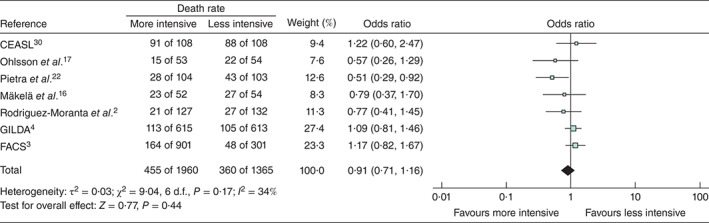
Forest plot showing odds ratios for death in seven randomized comparisons of more and less intensive follow-up. A Mantel–Haenszel random-effects model was used to produce an overall estimated odds ratio. Odds ratios are shown with 95 per cent confidence intervals

Among 7081 publications, there were 22 relevant articles^2–4,15–33^ describing 16 randomized comparisons (*Fig. 1*). Text summaries of all 16 randomized trials are provided in *Appendix S1* (supporting information). Five studies were excluded because there were no survival data available for analysis^28,31^ or they had high risk of bias^18,21,23^ (*Table 1*). The remaining 11 studies provided data on overall survival suitable for meta-analysis and, of these, seven^2–4,16–17,22,30^ included methods that allowed detection of metastases (*Table 2*, *Figs 2* and *3*). Two studies^20,27^ were confined to endoscopic examination following more or less intensive protocols (*Table 3*). Two studies^24,25^ followed the same protocol in each arm but were administered in hospital by a specialist or in a general practice setting.

**Table 1:** Studies excluded from meta-analysis

Trial	Start	End	Tests	No. of centres	No. of patients randomized	Reason for exclusion
Barillari *et al.*^18^	1980	1990	Colonoscopy	1	212	Inadequate survival data
Schoemaker *et al.*^21^	1984	1990	CT	1	325	Potential lack of allocation concealment
			Colonoscopy			
Secco *et al.*^23^	1988	1996	CEA	1	337	Reviewers could not reconcile conclusions with randomized groups
			Colonoscopy			
			Ultrasonography			
COLOFOL^28^	2006	2011	CEA	24	2571	Results not yet published
			CT			
CEAwatch^31^	2010	2012	CEA	11	3223	No outcome data reported

CEA, carcinoembryonic antigen.

**Table 2:** Details of the seven trials included in meta-analysis

Trial	Start	End	Tests^*^	No. of centres	No. of patients randomized	Authors' conclusion
CEASL^30^	1982	1993	CEA	58	216^†^	‘… highly unlikely that any survival advantage would be demonstrated for patients undergoing second-look surgery’
Ohlsson *et al.*^17^	1983	1986	Endoscopy	2	107	‘Intense follow-up … did not prolong survival in this study’
			CT			
			CEA			
Pietra *et al.*^22^	1987	1990	CEA	1	207	‘Our data support use of an intense follow-up plan after primary resection of large-bowel cancer, at least in patients with rectal cancer’
			Ultrasonography			
			CT			
			Chest X-ray			
			Colonoscopy			
Mäkelä *et al.*^16^	1988	1990	CEA	1	106	‘Earlier detection of recurrent carcinoma by intensified follow-up does not lead to increased re-resectability or improved 5-year survival’
			Chest X-ray			
			CT			
Rodriguez -Moranta *et al.*^2^	1997	2001	CEA	3	259	‘there was no difference in the probability of overall survival’
			Colonoscopy			
			CT			
			Ultrasonography			
			Chest X-ray			
GILDA^4^	1998	2006	CEA	41	1228	‘early diagnosis of cancer recurrence is not associated with overall survival benefit’
			Colonoscopy			
			Chest X-ray			
			Ultrasonography			
FACS^3^	2003	2009	CEA	39	1202	‘The number of deaths was not significantly different in the combined intensive monitoring groups *vs* the minimum follow-up group’
			CT			

* There were more tests, more frequent tests, or both in the group with more intensive monitoring.

† If the carcinoembryonic antigen (CEA) level was raised according to study criteria, patients were randomized to have this revealed to the clinical team or not.

**Table 3:** Details of single-centre trials confined to endoscopic methods of monitoring

Trial	Start	End	Tests	No. of patients	Authors' conclusions
Kjeldsen *et al.*^20^	1983	1994	Colonoscopy	597	‘no improvement in overall survival or in cancer-related survival’
Wang *et al.*^27^	1995	2001	Colonoscopy	326	There was higher detection of asymptomatic recurrence and more operations but the authors concluded that more intensive colonoscopy ‘did not improve overall survival’

Detailed protocols for investigations used for monitoring and their frequency are summarized in *Appendix S2* (supporting information).

### Quality of studies: risk of bias

Three studies were found to have a high risk of bias in at least one domain (*Table 4*). Blinding of participants and personnel (performance bias) was not possible and so there is a remaining risk of bias. Blinding of outcome assessment (detection bias) is not relevant for the main outcome measure, which is death/survival.

**Table 4:** Risk of bias

Reference	Randomization method	Allocation concealment	Incomplete outcome assessment	Selective reporting
CEASL^30^	Low	Low	Low	Low
Ohlsson *et al.*^17^	Unclear	Unclear	Low	Low
Pietra *et al.*^22^	Unclear	Unclear	Low	Low
Rodriguez-Moranta *et al.*^2^	Low	Low	Low	Low
Mäkelä *et al.*^16^	Unclear	Unclear	Low	Low
GILDA^4^	Unclear	Low	Low	Low
FACS^3^	Unclear	Unclear	Low	Low
Kjeldsen *et al.*^20^	Unclear	Unclear	High^*^	Low
Wang *et al.*^27^	Unclear	Low	Unclear	Low
Wattchow *et al.*^24^	Unclear	Low	Low	Low
Augestad *et al.*^25^	Low	Low	Low	Low
Schoemaker *et al.*^21^	Low	High^†^	Unclear	Low
Secco *et al.*^23^	High^‡^	Unclear	High^§^	Low
CEAwatch^31^	Low	Low	Low	Low

* Groups not balanced (290 : 307); drop-outs may not be included in assessment.

† Cards not in envelopes; groups not balanced (167 : 158).

‡ Method unclear; groups not balanced (108 : 84 and 84 : 61).

§ Patients excluded from survival rather than censored if lost to follow-up. COLOFOL has not reported so cannot be assessed; the methods of assignment were not described by Barillari *et al.*^18^, and this did not appear to be a true randomized comparison.

### Effectiveness of more intensive monitoring in advancing detection

For studies in which the time difference in detection was given (9 of 16) the advance in diagnosis was 2–30 months, with a median of 10 (i.q.r. 5–24) months (*Table 5*).

**Table 5:** Cancer recurrence rates and difference in time to detection in RCTs of monitoring strategies following potentially curative resection of colorectal cancer

			Recurrence (%)		Detection advance (months)
Reference	Recruitment	Methods tested^*^	Intensive	Control	
CEASL^30^	1982–1993	CEA	–^†^	–^†^	11
Ohlsson *et al.*^17^	1983–1986	CEA, CT, endoscopy			4
Kjeldsen *et al.*^20^	1983–1984	Endoscopy	26	26	9
Mäkelä *et al.*^16^	1988–1990	CT	42	39	5
Wang *et al.*^27^	1995–2001	Endoscopy	8	11	13
GILDA^4^	1998–2006	CT, endoscopy, liver ultrasonography	15	13	6
Wattchow *et al.*^24^	1998–2001	Setting: surgeon- or GP-led			2
FACS^3^					
CEA	2003–2009	CEA	22	14	24
CT	2004–2009	CT	20	14	30
CEA and CT	2005–2009	CEA, CT	17·5	14	24

* Only tests that were not the same in the two groups.

† Only a minority of patients meeting the stringent carcinoembryonic antigen (CEA) criteria were randomized, so detection was similar in both randomized groups and effectively 100 per cent.

### Main outcome measure: effectiveness in improving survival

The numbers of randomized patients and the numbers of all detection events and deaths are given in *Tables 6–8*. The principal result derives from the meta-analysis of HRs based on trials from which these could be estimated. The summary HR estimate was 0·98 (95 per cent c.i. 0·87 to 1·11), with no evidence of significant heterogeneity (*I*^2^ = 14 per cent) (*Fig. 2*).

**Table 6:** All-cause mortality rates in randomized trials

	All-cause mortality	
	Intensive follow-up	Less intensive follow-up
CEASL^30^	91 of 108 (84·3)	88 of 108 (81·5)
Mäkelä *et al.*^16^	23 of 52 (44)	27 of 54 (50)
Ohlsson *et al.*^17^	15 of 53 (28)	22 of 54 (41)
Kjeldsen *et al.*^20^	88 of 290 (30·3)	100 of 307 (32·6)
Pietra *et al.*^22^	28 of 104 (26·9)	43 of 103 (41·7)
Schoemaker *et al.*^21^	43 of 167 (25·7)	55 of 158 (34·8)
Secco *et al.*^23^	73 of 192 (38·0)	81 of 145 (55·9)
GILDA^4^	113 of 615 (18·4)	105 of 613 (17·1)
Rodriguez-Moranta *et al.*^2^	21 of 127 (16·5)	27 of 132 (20·5)
Wattchow *et al.*^24^	32 of 76 (42)	25 of 81 (31)
Wang *et al.*^27^	42 of 165 (25·5)	50 of 161 (31·1)
Augestad *et al.*^25^	1 of 55 (2)	4 of 55 (7)
FACS^3,34^	164 of 901 (18·2)	48 of 301 (15·9)
CEAwatch^31^	n.r.	n.r.
COLOFOL^28^	n.r.	n.r.
Total	734 of 2905 (25·3)	675 of 2272 (29·7)

Values in parentheses are percentages. n.r., Not reported.

**Table 7:** Local recurrence rates in randomized trials

	Local recurrence	
	Intensive follow-up	Less intensive follow-up
CEASL^30^	n.r.	n.r.
Mäkelä *et al.*^16^	12 of 52 (23)	11 of 54 (20)
Ohlsson *et al.*^17^	11 of 53 (21)	8 of 54 (15)
Kjeldsen *et al.*^19,20^	49 of 290 (16·9)	42 of 307 (13·7)
Pietra *et al.*^22^	20 of 104 (19·2)	26 of 103 (25·2)
Schoemaker *et al.*^21^	7 of 167 (4·2)	11 of 158 (7·0)
Secco *et al.*^23^	41 of 192 (21·4)	35 of 145 (24·1)
GILDA^4^	36 of 615 (5·9)	32 of 613 (5·2)
Rodriguez-Moranta *et al.*^2^	11 of 127 (8·7)	13 of 132 (10·1)
Wattchow *et al.*^24^	n.r.	n.r.
Wang *et al.*^27^	10 of 165 (6·1)	12 of 161 (7·5)
Augestad *et al.*^25^	6 of 55 (11)	8 of 55 (15)
FACS^3,34^	35 of 901 (3·9)	6 of 301 (2·0)
CEAwatch^31^	31 of 316 (9·8)	13 of 1182 (1·1)
COLOFOL^28^	n.r	n.r.
Total	269 of 3037 (8·9)	217 of 3265 (6·6)

Values in parentheses are percentages. n.r., Not reported.

**Table 8:** Distant recurrence rates in randomized trials

	Distant recurrence	
	Intensive follow-up	Less intensive follow-up
CEASL^30^	32 of 108 (29·6)	n.r.
Mäkelä *et al.*^16^	10 of 52 (19)	10 of 54 (19)
Ohlsson *et al.*^17^	9 of 53 (17)	12 of 54 (22)
Kjeldsen *et al.*^19,20^	34 of 290 (11·7)	48 of 307 (15·6)
Pietra *et al.*^22^	15 of 104 (14·4)	21 of 103 (20·4)
Schoemaker *et al.*^21^	n.r.	n.r.
Secco *et al.*^23^	38 of 192 (19·8)	47 of 145 (32·4)
GILDA^4^	59 of 615 (9·6)	42 of 613 (6·9)
Rodriguez-Moranta *et al.*^2^	20 of 127 (15·7)	19 of 132 (14·4)
Wattchow *et al.*^24^	n.r.	n.r.
Wang *et al.*^27^	n.r.	n.r.
Augestad *et al.*^25^	3 of 55 (5)	4 of 55 (7)
FACS^3,34^	39 of 901 (4·3)	18 of 301 (6·0)
CEAwatch^31^	n.r.	n.r.
COLOFOL^28^	n.r.	n.r.
Total	259 of 2497 (10·4)	221 of 1764 (12·5)

Values in parentheses are percentages. n.r., Not reported.

The meta-analysis of simple ORs for death based on the same seven studies is shown in *Fig. 3*; the summary OR for death was 0·91 (95 per cent c.i. 0·71 to 1·16). ORs were derived from the percentage of deaths in each arm at the time of reporting, whereas the HR gives an estimate of the overall relative survival, which is more relevant when considering a time-to-event endpoint.

**Fig. 4:**
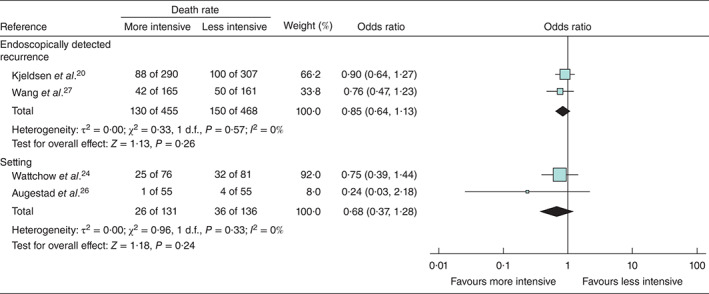
Forest plot for death in two studies in which the difference in monitoring was confined to endoscopy, and two studies for which the difference was between a hospital/specialist setting and a general practice setting. A Mantel–Haenszel random-effects model was used to produce an overall estimated odds ratio. Odds ratios are shown with 95 per cent confidence intervals

A meta-analysis of ORs for death is also shown for two studies in which the difference in monitoring was confined to endoscopy, and two studies for which the difference was between a hospital/specialist setting and a general practice setting (*Fig. 4*).

**Fig. 5:**
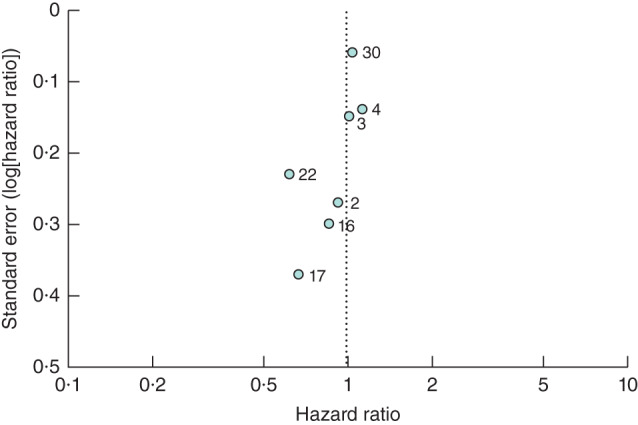
Funnel plot of studies included in meta-analysis. Reference numbers are shown

There is a residual possibility of publication bias as demonstrated in the asymmetry of the forest plot for the main analysis (*Fig. 2*) and the funnel plot (*Fig. 5*). Because of the proportion of the weight accredited by RevMan to CEASL (Carcino-Embryonic Antigen Second Look), and because in this study the monitoring was solely by CEA and not CT, a sensitivity analysis was performed after exclusion of CEASL. This did not alter the conclusion (HR 0·92, 0·76 to 1·36)

**Fig. 6:**
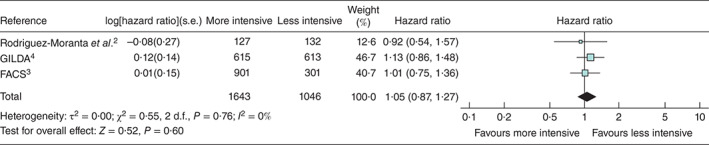
Subset analysis of three large multicentre RCTs published in 2006, 2014 and 2016, which included 80·9 per cent of all patients in the full systematic review. A random-effects inverse-variance model was used to produce an overall estimated hazards ratio. Hazard ratios are shown with 95 per cent confidence intervals

The three trials reporting from 2006 to 2016 were larger, multicentre, better quality studies and contributed 2689 (80·9 per cent) of the 3325 patients included in the overall meta-analysis (*Fig. 2*). The summary estimated HR from a meta-analysis of these trials was 1·05 (0·87 to 1·27) (*Fig. 6*).

## Discussion

The question addressed by this systematic review is whether follow-up strategies involving more intensive monitoring, with more frequent investigation and/or additional methods of detection, lead to an improvement in overall survival. Meta-analyses of the ORs and derived HRs from seven RCTs including 3325 patients showed no survival benefit from successively intensified monitoring policies. The authors of ten^2–4,16–17,20,24–25,27,30^ of the 11 trials reported no survival benefit from more intensive monitoring. This could have been due to lack of power, but the meta-analysis is consistent with the findings of individual studies. Three studies^4,24,26^ that reported outcomes on quality of life found no differences with respect to this outcome.

A random-effects model and, where possible, HRs were used to quantify outcomes, as the outcomes of interest occur over time. Owing to data limitations, ORs were used for some comparisons. Where it was possible to do both analyses, there was no difference in the conclusion. A limitation of this systematic review is that publication bias may have affected the observed outcomes as unpublished data, abstracts and presentations were not included. However, consideration of this possible bias would likely make a survival benefit even less plausible.

Intensive follow-up was reported to show significantly improved survival by the authors of only one study^22^. It was attributed to reappearance of treatable residual disease after resection of rectal (as opposed to colonic) cancer. In exploratory subset analyses, other studies^2,4^ showed a survival difference in favour of more intensive monitoring benefit where local recurrence of rectal cancer was found endoscopically.

Earlier meta-analyses^1,35^ suggested a favourable effect on survival. This was not found in the present meta-analysis, which included larger and methodologically more robust trials reported in the past couple of years. These showed poorer survival in the more intensively screened groups, although the results were not significant individually. The survival results of this updated meta-analysis show no benefit. Although not statistically significant, the point estimates consistently suggest an adverse effect on survival. What these later RCTs have in common is that they were multicentre studies run from trial centres. It is possible that the commitment of physicians involved in follow-up has made a difference to outcomes in smaller institutional studies. Many of these patients will have had individualized treatment including systemic chemotherapy, but no overall benefit from monitoring and earlier detection has been shown in the meta-analysis. This analysis gives a coherent and trustworthy, but disappointingly negative, message about the hoped-for survival benefit of intensification of active monitoring after primary resection of colorectal cancer.

## Supplementary Material

bjs10233-sup-0001-AppendixS1 **Appendix S1.** Text summaries of 16 randomized trials

bjs10233-sup-0002-AppendixS2 **Appendix S2.** Spreadsheet of all monitoring methods (Excel spreadsheet)
