# Supplementary material for: Meta-analysis of colorectal cancer follow-up after potentially curative resection
Source: Br J Surg. 2016 Aug 4;103(10):1259–68. doi: 10.1002/bjs.10233 (PMC5031212; doi:10.1002/bjs.10233)
Supplement: bjs10233-sup-0001-AppendixS1 — Appendix S1. Text summaries of 16 randomized trials [file bjs10233-sup-0001-appendixs1.docx]

**BJS10233**

**Meta-analysis of colorectal cancer follow-up after potentially curative resection**

S. Mokhles, F. Macbeth, V. Farewell, F. Fiorentino, N. Williams, R. N. Younes, J. J. M Takkenberg and T. Treasure

**Appendix S1** Text summaries of 16 randomized trials

1. **Seven randomized trials with overall survival data extractable for hazard ratio random-effects models in date order of the trials opening**From 1982 to 1993, the Carcino-Embryonic Antigen Second Look (CEASL) trial recruited 1447 patients and randomized 216 in 58 UK hospitals. Patients had monthly CEA assays and the 216 who had an increased level according to the protocol were allocated randomly to have it revealed and acted upon or concealed^30^. Conclusion: ‘it was highly unlikely that any survival advantage would be demonstrated for patients undergoing second-look surgery.’ We have access to the archived trial data.
2. From 1983 to 1986, the Departments of Surgery of Lund University and Helsinborg Hospital, Sweden, randomized 107 patients. The more actively monitored group were seen 13 times in the first 5 years and had sigmoidoscopy and chest X-ray on each occasion and CT and colonoscopy on four or five occasions (*Appendix S2*, supporting information). The median follow-up time was 6.8 (range 5.5–8.8) years. The authors concluded ‘Intense follow-up did not prolong survival in this study’^17^. We have had no reply to our enquiry for further information dated 8 September 2015.
3. From 1987 to 1990, Pietra and colleagues^22^ at the Institute of General Surgery, University of Parma, Italy, randomized 207 patients. Patients in the intensive arm were seen 14 times in the first 5 years and had clinical examination, CEA and ultrasonography at every visit, and annual CT, chest X-ray and colonoscopy. Control patients were seen six times and had CEA assay, chest X-ray and colonoscopy annually and no CT (*Appendix S2*, supporting information). Both patient groups had complete follow-up beyond 5 years. The authors concluded ‘our data support use of an intense follow-up plan after primary resection of large-bowel cancer, at least in patients with rectal cancer’. This was because endoscopic detection of rectal cancer recurrence accounted for the improved overall survival. We have established communication with the department. The senior author died 15 years ago and there is unlikely to be any record available.
4. From 1988 to 1990, Mäkelä and colleagues^15,16^ in the Department of Surgery at Oulo University Hospital, Finland, randomized 106 patients to either an intensified or conventional follow-up. All patients were seen 14 times in 5 years and had CEA, chest X-ray and other tests. In the intensive group ultrasonography was performed every 6 months, and CT and colonoscopy annually. (*Appendix S2*, supporting information). All patients were followed up for more than 5 years. The authors concluded that ‘earlier detection of recurrent carcinoma by intensified follow-up does not lead to increased re-resectability or improved 5-year survival’. We have had as yet no reply to our letter dated 8 September 2015 to establish communication with the trialists.
5. From 1997 to 2001, in three Catalan hospitals in Barcelona, Terrassa and Vic, 259 patients were randomized to either a simple strategy of CEA monitoring 14 times in 5 years and colonoscopy at 1 and 3 years, or an intensive strategy in which were added annual chest X-ray, CT or liver ultrasound imaging (for rectal and colonic cancer respectively) 6 monthly for two years and then annually, and colonoscopy increased to annually^2^. (*Appendix S2*, supporting information). The median follow-up was 48 months. The authors concluded ‘there was no difference in the probability of overall survival’ at 48 months but subsets of stage II and rectal tumours had higher survival with an intensive strategy. We are in correspondence with the senior author, who confirms that there was no difference in survival overall, but for the rectal group there were higher detection and survival rates attributable to endoscopy (personal communication, 21 November 2015).
6. From 2003 to 2009, the FACS trial recruited in 39 National Health Service hospitals in the UK^3^. 1202 patients were randomly assigned to CEA monitoring, CT, both or neither with mean(s.d.) follow-up 3.78(1.53), 3.69(1.59), 3.74(1.58) and 3.64(1.69) years respectively. The authors concluded that the ‘number of deaths was not significantly different in the combined intensive monitoring groups *versus* minimum follow-up’. We are in communication with the Chief Investigator,
7. From 1998 to 2006, a multicentre study (GILDA) randomized 1228 evaluable patients in 39 centres in Italy and one each in Spain and the USA^4,33^. All patients had 11 hospital visits and CEA assay in the first 5 years. Intensively monitored patients had colonoscopy, chest X-ray, liver ultrasonography and CT on five to 11 occasions, compared with one or two of the other investigations and no CT (*Appendix S2*, supporting information). The diagnosis of recurrence was advanced by a mean difference of 6 months but the authors concluded that ‘early diagnosis of cancer recurrence is not associated with overall survival benefit’. There was no difference in quality of life between the two arms of the trial. We are in communication with the leaders of the trial.

**Studies confined to endoscopic methods of monitoring**

1. From 1983 to 1994, the Departments of Surgery and Pathology of Odense University Hospital, Denmark, randomized 597 patients^19,20^. The more intensively monitored group were seen eight times in the 5 years after surgery and had clinical examination, colonoscopy, chest X-ray and other investigations *versus* one examination at 5 years in the control group (*Appendix S2*, supporting information). Follow-up for more than 5 years was possible in 310 patients. The authors concluded that detection was earlier, more operations were performed but that there was ‘no improvement in overall survival, or cancer-related survival’. There was considered to be a high risk of bias for incomplete outcome assessment, and it was excluded from the sensitivity analysis. We received a reply (20 September 2015) from the second author who has been retired for 11 years and no more information is available.
2. From 1995 to 2001, in Sun Yat-sen University Hospital, Guanzhou, China, 326 patients were randomized between two colonoscopy strategies entailing 18 examinations over 5 years *versus* three investigations^27^ (*Appendix S2*, supporting information). The median follow-up was 74 (range 10–129) months in the intensive follow-up group and 69 (range 8–134) months in the routine follow-up group. There was greater detection of asymptomatic recurrence and more operations, but the authors concluded that more intensive colonoscopy ‘did not improve overall survival’. We have had as yet no reply to our letter dated 8 September 2015 to establish communication with the trialists.

**Studies in which the randomization was to hospital *versus* general practice clinical setting**

1. From 1998 to 2001, 14 hospitals in four Australian states randomized 203 patients (of 611 assessed and 340 eligible) to follow-up by general practitioners or surgeons^24^. The follow-up time was 2 years. The authors concluded that patients experienced similar outcomes. There were no differences in Hospital Anxiety or Depression scores, or SF-12^®^. We have had as yet no reply to our letter dated 8 September 2015 to establish communication with the trialists
2. From 2007 to 2011, a multicentre trial in Norway (4 trusts, 11 hospitals, 88 local communities) recruited 110 patients to either follow-up by hospital surgeons or community general practitioners^25,26^. The follow-up time in the hospital surgeons group was 903 months and 897 months in the group of community general practitioners. The authors concluded that there was no difference in detection or survival. There were no differences in patient-reported outcomes (European Organization for Research and Treatment of Cancer QLQ C-30 or EQ-5D™). There were fewer hospital visits in the general practice follow-up but it is not clear whether patient preferences were captured at the time of reviewing. We are in ongoing communication with the first author.

**Studies excluded from meta-analysis**

1. From 1980 to 1990, in the First Department of Surgery, University of Rome ‘La Sapienza’, Italy, 212 patients were randomized to have colonoscopy at 1- or 2-year intervals^18^. Mean follow-up per patient was 62 (range 37–142) months. The authors found no difference in the number or stage of anastomotic recurrences detected. There were inadequate data on survival and so this study was not included in meta-analysis. We have had as yet no reply to our letter dated 8 September 2015 to establish communication with the trialists.
2. From 1984 to 1990, Flinders Medical Centre and Repatriation Hospital, Adelaide, Australia, randomized 325 patients. Patients in both standard and intensive follow-up groups had clinical review, liver function tests, CEA and faecal occult blood tests 3 monthly for 2 years, and thereafter 6 monthly for 5 years or until a major endpoint was reached. Patients in the intensive arm of the trial in addition underwent yearly chest X-ray, CT of the liver, and colonoscopy. Patients in the standard group had these investigations only if indicated on clinical or screening test abnormality, and after completing 5 years of follow-up. Patients in the intensive arm had yearly chest X-ray, liver CT and colonoscopy^21^. Control patients had these investigations at 5 years or if indicated clinically (*Appendix S2*, supporting information). All patients were followed up for a minimum of 5 years or until death. The study was excluded from meta-analysis after sensitivity analysis because there was considered to be a high risk of bias due to potential lack of allocation concealment^6^. We have been in communication with Dr Schoemaker in line with published recommendations^7^. Despite the authors’ published conclusion that the addition of annual chest X-ray, CT and colonoscopy ‘will not improve survival from colorectal cancer when added to symptom and simple screening review’^21^ there is an expectation that these investigations will be done.
3. From 1988 to 1996, surgeons at University of Genoa School of Medicine, Italy, prospectively divided 358 patients into high-risk (200) and low-risk (158) groups^23^. They were then randomized into subgroups: 192 for ‘risk adapted’ follow-up, 145 to minimal surveillance, and 21 dropped out (*Appendix S2*, supporting information). Median follow-up was 61.5 and 42 months for patients at high risk and at low risk respectively. The study was initially included in the review but the adjudicator considered there was high risk of bias in the method of randomization and incompleteness of outcome assessment. The data were excluded from the sensitivity analysis. The authors’ conclusion was that ‘risk-adapted follow-up has significantly improved the targeting of curative reoperations and overall survival of patients …’ a conclusion which we could not verify as being derived from randomized patient outcomes. We have had as yet no reply to our letter dated 8 September 2015 to establish communication with the trialists.
4. From 2010 to 2012, the CEAwatch trial recruited 3223 patients in 11 hospitals in the Netherlands in a trial which sequentially changed in a stepped wedge design to an intensified follow-up of CEA measurements every 2 months with imaging in the case of two rises^31,32^. The authors reported that the time to detection was shorter and that a ‘significantly higher proportion of recurrences can be treated with curative intent’. There are no survival data reported so far. We are in communication with the first author.
5. COLOFOL^28,29^. From 2006 to 2011, a multicentre trial in 15 Swedish, eight Danish and one Uruguayan centre randomized 2571 patients to CT and CEA five times *versus* twice in the first 3 years. The authors reported that there was no difference in survival at the European Society of Coloproctology meeting (Dublin, Ireland; 23 September 2015). We are in communication with the first author who has shared the Kaplan–Meier plot for overall survival. The results have not yet been published and we have no data to include in our meta-analysis.

**Reference list from main paper** (not all are cited in this appendix)

1 Renehan AG, Egger M, Saunders MP, O’Dwyer ST. Impact on survival of intensive follow up after curative resection for colorectal cancer: systematic review and meta-analysis of randomised trials. *BMJ* 2002; **324**: 813.

2 Rodriguez-Moranta F, Saló J, Arcusa A, Boadas J, Piñol V, Bessa X *et al.* Postoperative surveillance in patients with colorectal cancer who have undergone curative resection: a prospective, multicenter, randomized, controlled trial. *J Clin Oncol* 2006; **24**: 386–393.

3 Primrose JN, Perera R, Gray A, Rose P, Fuller A, Corkhill A, George S, Mant D. Effect of 3 to 5 years of scheduled CEA and CT follow-up to detect recurrence of colorectal cancer: the FACS randomized clinical trial. *JAMA* 2014; **311**: 263–270.

4 Rosati G, Ambrosini G, Barni S, Andreoni B, Corradini G, Luchena G *et al.*; A randomized trial of intensive *versus* minimal surveillance of patients with resected Dukes B2-C colorectal carcinoma. *Ann Oncol* 2016; **27**: 274–280.

5 Moher D, Liberati A, Tetzlaff J, Altman DG. Preferred reporting items for systematic reviews and meta-analyses: the PRISMA statement. *BMJ* 2009; **339**: b2535.

6 Higgins JP, Altman DG, Gotzsche PC, Juni P, Moher D, Oxman AD *et al.* The Cochrane Collaboration’s tool for assessing risk of bias in randomised trials. *BMJ* 2011; **343**: d5928.

7 Roberts I, Ker K, Edwards P, Beecher D, Manno D, Sydenham E. The knowledge system underpinning healthcare is not fit for purpose and must change. *BMJ* 2015; **350**: h2463.

8 Williamson PR, Smith CT, Hutton JL, Marson AG. Aggregate data meta-analysis with time-to-event outcomes. *Stat Med* 2002; **21**: 3337–3351.

9 Parmar MK, Torri V, Stewart L. Extracting summary statistics to perform meta-analyses of the published literature for survival endpoints. *Stat Med* 1998; **17**: 2815–2834.

10 Tierney JF, Stewart LA, Ghersi D, Burdett S, Sydes MR. Practical methods for incorporating summary time-to-event data into meta-analysis. *Trials* 2007; **8**: 16.

11 Higgins JP, Thompson SG, Deeks JJ, Altman DG. Measuring inconsistency in meta-analyses. *BMJ* 2003; **327**: 557–560.

12 Barton MK. Metastasectomy on the rise across several cancer types. *CA Cancer J Clin* 2015; **65**: 163–164.

13 Bartlett EK, Simmons KD, Wachtel H, Roses RE, Fraker DL, Kelz RR *et al.* The rise in metastasectomy across cancer types over the past decade. *Cancer* 2015; **121**: 747–757.

14 Jawed I, Wilkerson J, Prasad V, Duffy AG, Fojo T. colorectal cancer survival gains and novel treatment regimens: a systematic review and analysis. *JAMA Oncol* 2015; **1**: 787–795.

15 Makela J, Laitinen S, Kairaluoma MI. Early results of follow-up after radical resection for colorectal cancer. Preliminary results of a prospective randomized trial. *Surg Oncol* 1992; **1**: 157–161.

16 Mäkelä JT, Laitinen SO, Kairaluoma MI. Five-year follow-up after radical surgery for colorectal cancer. Results of a prospective randomized trial. *Arch Surg* 1995; **130**: 1062–1067.

17 Ohlsson B, Breland U, Ekberg H, Graffner H, Tranberg KG. Follow-up after curative surgery for colorectal carcinoma. Randomized comparison with no follow-up. *Dis Colon Rectum* 1995; **38**: 619–626.

18 Barillari P, Ramacciato G, Manetti G, Bovino A, Sammartino P, Stipa V. Surveillance of colorectal cancer: effectiveness of early detection of intraluminal recurrences on prognosis and survival of patients treated for cure. *Dis Colon Rectum* 1996; **39**: 388–393.

19 Kjeldsen BJ, Kronborg O, Fenger C, Jørgensen OD. The pattern of recurrent colorectal cancer in a prospective randomised study and the characteristics of diagnostic tests. *Int J Colorectal Dis* 1997; **12**: 329–334.

20 Kjeldsen BJ, Kronborg O, Fenger C, Jørgensen OD. A prospective randomized study of follow-up after radical surgery for colorectal cancer. *Br J Surg* 1997; **84**: 666–669.

21 Schoemaker D, Black R, Giles L, Toouli J. Yearly colonoscopy, liver CT, and chest radiography do not influence 5-year survival of colorectal cancer patients. *Gastroenterology* 1998; **114**: 7–14.

22 Pietra N, Sarli L, Costi R, Ouchemi C, Grattarola M, Peracchia A. Role of follow-up in management of local recurrences of colorectal cancer: a prospective, randomized study. *Dis Colon Rectum* 1998; **41**: 1127–1133.

23 Secco GB, Fardelli R, Gianquinto D, Bonfante P, Baldi E, Ravera G *et al.* Efficacy and cost of risk-adapted follow-up in patients after colorectal cancer surgery: a prospective, randomized and controlled trial. *Eur J Surg Oncol* 2002; **28**: 418–423.

24 Wattchow DA, Weller DP, Esterman A, Pilotto LS, McGorm K, Hammett Z. General practice *vs* surgical-based follow-up for patients with colon cancer: randomised controlled trial. *Br J Cancer* 2006; **94**: 1116–1121.

25 Augestad KM, Vonen B, Aspevik R, Nestvold T, Ringberg U, Johnsen R. Should the surgeon or the general practitioner (GP) follow up patients after surgery for colon cancer? A randomized controlled trial protocol focusing on quality of life, cost-effectiveness and serious clinical events. *BMC Health Serv Res* 2008; **8**: 137.

26 Augestad KM, Norum J, Dehof S, Aspevik R, Ringberg U, Nestvold T *et al.* Cost-effectiveness and quality of life in surgeon *versus* general practitioner-organised colon cancer surveillance: a randomised controlled trial. *BMJ Open* 2013; **3**: pii: e002391.

27 Wang T, Cui Y, Huang WS, Deng YH, Gong W, Li CJ. The role of postoperative colonoscopic surveillance after radical surgery for colorectal cancer: a prospective, randomized clinical study. *Gastrointest Endosc* 2009; **69**: 609–615.

28 Wille-Jørgensen P, Laurberg S, Påhlman L, Carriquiry L, Lundqvist N, Smedh K *et al.* An interim analysis of recruitment to the COLOFOL trial. *Colorectal Dis* 2009; **11**: 756–758.

29 Hansdotter Andersson P, Wille-Jørgensen P, Horváth-Puhó E, Petersen SH, Martling A, Sørensen HT *et al.* The COLOFOL trial: study design and comparison of the study population with the source cancer population. *Clin Epidemiol* 2016; **8**: 15–21.

30 Treasure T, Monson K, Fiorentino F, Russell C. The CEA Second-Look Trial: a randomised controlled trial of carcinoembryonic antigen prompted reoperation for recurrent colorectal cancer. *BMJ Open* 2014; **4**: e004385.

31 Verberne CJ, Zhan Z, van den Heuvel E, Grossmann I, Doornbos PM, Havenga K *et al.* Intensified follow-up in colorectal cancer patients using frequent carcino-embryonic antigen (CEA) measurements and CEA-triggered imaging: results of the randomized ‘CEAwatch’ trial. *Eur J Surg Oncol* 2015; **41**: 1188–1196.

32 Verberne CJ, Wiggers T, Grossmann I, de Bock GH, Vermeulen KM. Cost-effectiveness of a Carcinoembryonic Antigen (CEA)-based follow-up programme for colorectal cancer (the CEAwatch trial). *Colorectal Dis* 2016; **18**: O91-6.

33 Grossmann EM, Johnson FE, Virgo KS, Longo WE, Fossati R: Follow-up of colorectal cancer patients after resection with curative intent – the GILDA trial. *Surg Oncol* 2004; **13**: 119–124.

34 Pugh SA, Shinkins B, Fuller A, Mellor J, Mant D, Primrose JN. Site and stage of colorectal cancer influence the likelihood and distribution of disease recurrence and postrecurrence survival: data from the FACS randomized controlled trial. *Ann Surg* 2016; **263**: 1143–1147.

35 Jeffery GM, Hickey BE, Hider P. Follow-up strategies for patients treated for non-metastatic colorectal cancer. *Cochrane Database Syst Rev* 2002; (1)CD002200.
